# Supplementary material for: HIVprotI: an integrated web based platform for prediction and design of HIV proteins inhibitors
Source: J Cheminform. 2018 Mar 9;10:12. doi: 10.1186/s13321-018-0266-y (PMC5845081; doi:10.1186/s13321-018-0266-y)
Supplement: Supplementary file 2 — Additional file 2. Source code of HIVProtI web server. [file 13321_2018_266_MOESM2_ESM.zip › HIVprotI_Source-code/hivprotI/weka-3-6-6/doc/weka/core/matrix/LinearRegression.html]

LinearRegression


---


|  |  |  |
| --- | --- | --- |
| **Overview** \| **Package** \| **Class** \| **Tree** \| **Deprecated** \| **Index** \| **Help** \| **Weka's home** | |  |
| **PREV CLASS**   **NEXT CLASS** | **FRAMES**    **NO FRAMES**     **All Classes** |  |
| SUMMARY: NESTED \| FIELD \| CONSTR \| METHOD | DETAIL: FIELD \| CONSTR \| METHOD |  |


---


## weka.core.matrix Class LinearRegression

```
java.lang.Object
  weka.core.matrix.LinearRegression
```

**All Implemented Interfaces:**: RevisionHandler

---

``` public class LinearRegression extends java.lang.Object implements RevisionHandler ```

Class for performing (ridged) linear regression.

**Version:**
:   $Revision: 1.4 $

**Author:**
:   Fracpete (fracpete at waikato dot ac dot nz)

---

**Constructor Summary**

`LinearRegression(Matrix a,
Matrix y,
double ridge)`
  
          Performs a (ridged) linear regression.

`LinearRegression(Matrix a,
Matrix y,
double[] w,
double ridge)`
  
          Performs a weighted (ridged) linear regression.


| **Method Summary** | |
| --- | --- |
| `double[]` | `getCoefficients()`             returns the calculated coefficients |
| `java.lang.String` | `getRevision()`             Returns the revision string. |
| `java.lang.String` | `toString()`             returns the coefficients in a string representation |

**Methods inherited from class java.lang.Object**

`equals, getClass, hashCode, notify, notifyAll, wait, wait, wait`

**Constructor Detail**

### LinearRegression

```
public LinearRegression(Matrix a,
                        Matrix y,
                        double ridge)
```

:   Performs a (ridged) linear regression.

    **Parameters:**: `a` - the matrix to perform the regression on: `y` - the dependent variable vector: `ridge` - the ridge parameter **Throws:**: `java.lang.IllegalArgumentException` - if not successful

---


### LinearRegression

```
public LinearRegression(Matrix a,
                        Matrix y,
                        double[] w,
                        double ridge)
```

:   Performs a weighted (ridged) linear regression.

    **Parameters:**: `a` - the matrix to perform the regression on: `y` - the dependent variable vector: `w` - the array of data point weights: `ridge` - the ridge parameter **Throws:**: `java.lang.IllegalArgumentException` - if the wrong number of weights were provided.


**Method Detail**

### getCoefficients

```
public final double[] getCoefficients()
```

:   returns the calculated coefficients

    :   **Returns:**: the coefficients

---


### toString

```
public java.lang.String toString()
```

:   returns the coefficients in a string representation

    :   **Overrides:**: `toString` in class `java.lang.Object`

---


### getRevision

```
public java.lang.String getRevision()
```

:   Returns the revision string.

    :   **Specified by:**: `getRevision` in interface `RevisionHandler`
    :   **Returns:**: the revision


---


|  |  |  |
| --- | --- | --- |
| **Overview** \| **Package** \| **Class** \| **Tree** \| **Deprecated** \| **Index** \| **Help** \| **Weka's home** | |  |
| **PREV CLASS**   **NEXT CLASS** | **FRAMES**    **NO FRAMES**     **All Classes** |  |
| SUMMARY: NESTED \| FIELD \| CONSTR \| METHOD | DETAIL: FIELD \| CONSTR \| METHOD |  |


---
